# Sleep homeostasis impairment across obstructive sleep apnea severity: findings from a population-based cohort study

**DOI:** 10.1007/s44470-026-00131-6

**Published:** 2026-08-04

**Authors:** Rama Maganti, Jun Wang, Scott Hetzel

**Affiliations:** 1 Department of Neurology, University of Wisconsin School of Medicine and Public Health, 1685 Highland Ave, Madison, WI 53706 USA; 2 Department of Biostatistics and Medical Informatics, University of Wisconsin School of Medicine and Public Health, WARF Office Building, Room 207G, 610 Walnut Street, Madison, WI 53726 USA

**Keywords:** Obstructive sleep apnea, Slow wave activity, Slope of slow wave, Sleep homeostasis

## Abstract

**Purpose:**

Obstructive sleep apnea (OSA) is a sleep disorder that causes recurrent airway obstruction and sleep fragmentation and increases cardiometabolic, cognitive, and mortality risk. Although slow-wave activity (SWA) and sleep homeostasis (SH) are central to restorative sleep and synaptic regulation, the extent to which SH is disrupted across OSA severity remains unclear.

**Methods:**

In this population-based cohort study, we analyzed 945 polysomnography (PSG) studies from adults with mild, moderate, severe OSA, or no OSA. Sleep parameters including apnea–hypopnea index (AHI) and slow wave activity (SWA 0.5–4 Hz) from electroencephalography (EEG) in non-rapid eye movement (NREM) sleep were extracted. SH was assessed with overnight decay of SWA, slopes of slow waves, and change in SWA with wake after sleep onset (WASO). Liner regression models were used to identify independent predictors of SH measures.

**Results:**

SWA decay across successive NREM episodes was attenuated in severe OSA group. Higher AHI (*p* < 0·001) and greater WASO (*p* < 0·001) were associated with a flatter decay of SWA. For a given level of SWA decay, males exhibited higher AHI than females (*p* < 0·0001). Slope of slow waves decreased from the first to last hour of NREM sleep in all groups (*p* < 0.01) except severe OSA. Multivariable models revealed that diabetes, use of antidepressant, anti-anxiety, antihypertensive, and sedative medications and high caffeine intake independently predicted impaired SH, whereas greater N3 sleep percentage was associated with preserved SWA decline.

**Conclusion:**

These data demonstrate SH is disrupted in OSA but additionally modulated by metabolic and pharmacologic factors. Targeting SH may represent a strategy to mitigate neurocognitive and physiological consequences of OSA.

**Brief Summary:**

Obstructive sleep apnea (OSA) is associated with adverse health outcomes including hypertension, cardiovascular complications, cognitive decline as well as mortality.

Impaired sleep homeostasis in OSA may represent a mechanistic link between sleep-disordered breathing and adverse health outcomes as slow wave activity is critical for synaptic downscaling, memory consolidation, and metabolic regulation.

OSA is known to impair sleep homeostasis though it is unknown if OSA severity matters. Our data suggested that severe OSA impaired sleep homeostasis but clinical factors should be considered as they can impact sleep homeostasis as well.

Future research should evaluate whether therapeutic interventions for OSA can reverse all these impairments and whether treatments should also be directed towards correcting sleep homeostasis impairment as well.

**Supplementary Information:**

The online version contains supplementary material available at 10.1007/s44470-026-00131-6.

## Introduction

Obstructive sleep apnea (OSA) is associated with long-term cardiovascular and metabolic morbidity, impaired cognitive function, and increased mortality [1–5]. Importantly, apnea–hypopnea index (AHI) and oxygen desaturation index (ODI) do not fully capture disease burden. Growing evidence suggests that cumulative hypoxic burden (depth and duration of desaturations), reduced N3 sleep, REM-predominant OSA, sleep fragmentation from recurrent arousals, and nocturnal autonomic dysregulation independently contribute to adverse cardiovascular and neurocognitive outcomes [6–9].

Sleep is a fundamental physiologic process in humans and is regulated by coupled circadian and homeostatic processes. The circadian process modulates sleep propensity throughout the 24 h, whereas sleep homeostasis (SH) is a process where sleep pressure accumulates during prior wakefulness and dissipates during subsequent sleep [10]. SH is typically quantified by the power in delta activity or slow wave activity (SWA; 0.5–4 Hz) during non-rapid eye movement (NREM) sleep, in electroencephalogram (EEG) or local field potentials (LFPs) [11, 12]. Although preceding sleep–wake history is considered a key determinant of sleep amount and intensity, the circadian process also plays an important role [11]. According to synaptic homeostasis hypothesis, sleep pressure reflects the strength of the synapses which increase with wakefulness and downscales during sleep. Thus, sleep pressure and synaptic potentiation are consequences of wakefulness, and sleep homeostasis serves to downscale synapses and reduce sleep pressure [13]. In addition, SH serves to maintain energy balance [12, 13] and cognition [14, 15] and supports immune function [16].

Experimentally, SH is assessed mainly by measuring an increase in SWA in humans and animals after sleep deprivation [17]. A second SH measure is the overnight dynamics of SWA, where it is highest at the beginning of the night, gradually dissipating in successive NREM episodes across the night [18]. Other measures include amplitude, frequency, and slopes of the slow waves which are all higher at the beginning of the night and lower in the end of the night [17, 18]. Since SWA homeostatically increases with time awake, increase in SWA with wake after sleep onset (WASO) time is also expected. This was previously demonstrated in rodents as well as humans [19, 20] and hence is another measure of SH.

SH is known to be impaired in OSA and neurological disorders such as Alzheimer’s disease [21, 22] and epilepsy [23]. There is a dearth of studies examining SH in OSA. There was one study which showed the exponential decay of SWA being slower in patients with mild sleep apnea compared to controls without OSA [24]. There are no studies that have examined all measures of SH systematically in large cohorts. We hypothesized that increasing OSA severity is associated with progressive impairment of SH, reflected by blunted overnight SWA decay, reduced overnight decline in slope of slow waves, and altered SWA responses to sleep fragmentation or WASO time. We evaluated whether SH measures are impacted by OSA severity, sleep fragmentation, clinical variables in patients.

## Methods

Participant data utilized were from the Wisconsin Sleep Cohort under which participants provided informed consent with the study protocols approved by the University of Wisconsin, School of Medicine Institutional Review Board. Employees of the state of Wisconsin were invited as participants. Those who agreed to participate underwent 1 to 4 polysomnography studies between 1988 and 2015. A total of 1520 eligible participants underwent at least 1 polysomnogram. From these, studies from 945 participants were analyzed, all of which were the first study each participant had.

### Procedures

Data on BMI, medical history, medication use, alcohol use, smoking habits, self-reported sleep habits, and daytime sleepiness using Epworth Sleepiness Scale were collected. Polysomnography was recorded according to methods described previously [25]. Sleep state and respiratory event scoring were performed by trained sleep technicians using standard criteria for sleep stages and for obstructive apneas or hypopneas, the American Academy of Sleep Medicine criteria of 3% desaturation was used. Variables collected from sleep studies included a number of sleep parameters such as sleep efficiency, time spent in vigilance states, wake after sleep onset (WASO), apnea–hypopnea index (AHI), and oxygen desaturation index (ODI).

### Sleep homeostasis measures

**Fig. 1:**
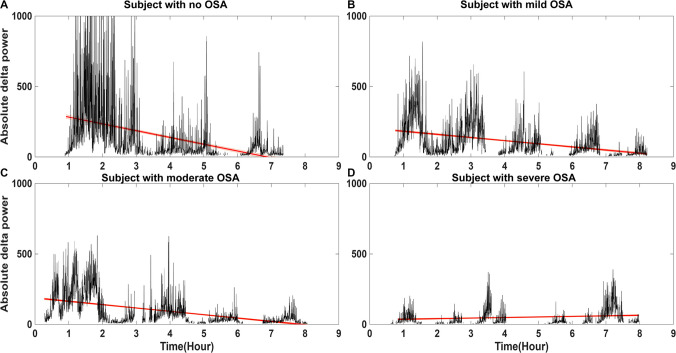
Panels show absolute delta power during NREM sleep. **A** Patient with no OSA (R2 0.18; Slope − 48.12); **B** patient with mild OSA (R2 0.17; slope − 22.23); **C** patient with moderate OSA (R2 0.26; Slope − 23.80); **D** patient with severe OSA ((R2 0.02; slope 3.98). The solid red line in each panel is the fitted linear regression of mean delta activity in 30 s epoch. The dotted lines represent 95% confidence interval for the regression fit

Power in the delta band (0.5–4 Hz) was extracted from the frontal and central leads of the EEG that was converted into a European Data Format (EDF). EEG sampled at 100 Hz was band pass filtered between 0.5 and 30 Hz. The absolute power in the delta band (0.5–2 Hz or 0.5–4 Hz) was extracted from each 6-s epoch of N2 and N3 sleep in the EDFs using the Welch Function in Python (Scipy package) for each individual (examples for four individual participants are shown in Fig. 1). The absolute delta power was then normalized to account for interindividual variability by dividing delta with the sum of all other frequency bands including theta (5–7 Hz), alpha (8–13 Hz), beta (14–22 Hz), and sigma (> 22 Hz) to extract normalized SWA from manually sleep scored record (here after referred to as SWA).

First SH measure is change in SWA across NREM clusters. Most participants had 4 NREM clusters per night and only a small minority had > 5 clusters. As a result, we limited the analysis to 4 NREM clusters. Each NREM episode that is composed of less than 5 min of wake was categorized as a “NREM cluster.” Decay of SWA was correlated with AHI/apnea severity. A steep decay of SWA across the night or in successive NREM clusters indicates intact sleep homeostasis, with efficient dissipation of sleep pressure early in sleep. In contrast, a slower, flatter, or more dispersed decay reflects impaired or inefficient dissipation of sleep pressure, suggesting disruption of homeostatic regulation.

The second SH measure was slope of slow waves. After raw EEG was band pass filtered between 0.5 and 4 Hz, delta band activity was identified from N2 and N3 sleep. Slope of slow wave were defined by the downslope of the negative half-wave, similar to other studies [17]. We applied a 50-ms moving average filter to automatically detect zero-crossings on the signal and select the descending segments of negative half-waves whose zero-crossings were separated by 0.25 to 1 s. Subsequently, we identified negative peaks (troughs) of slow waves after each zero-crossing on the signal using an automated algorithm. As a final step, we divided the amplitude difference (zero-crossing to negative peak in µV) by their interval. We also computed the slopes of all slow waves identified in the first and last hours of NREM sleep during the night of the study. A decline in slow wave slope from early to late NREM sleep represents normal homeostatic downscaling of synaptic strength. Failure of slopes to decrease across the night suggests impaired sleep homeostasis and reduced synaptic renormalization.

A final measure of SH increased in SWA with WASO, where mean SWA was examined as a function of cumulative WASO duration across OSA groups. A significant increase in wakefulness after sleep onset (WASO) should transiently increase sleep pressure reflected in subsequent compensatory increase in SWA during subsequent NREM sleep. A lack of such increase proportional to the time in WASO is indicative of disruption of normal SH.

### Statistical analysis

The clinical and polysomnography variable differences between the four groups were compared with one-way ANOVA. To quantify the overnight decline in SWA, normalized SWA was computed for artifact-free NREM epochs and plotted against time (hours). A linear regression model estimated the decay of SWA across the night for each group.

Demographic and clinical variables were compared by OSA severity group using Fisher’s exact, Chi-square, ANOVA, or Kruskal–Wallis tests when appropriate for the distribution of the variable. Data were summarized by *N* (%), mean (SD), or median (interquartile range-IQR) based on the distribution of the variable. Decay of SWA across NREM clusters was assessed using two-way ANOVA with repeated measures (within-subject factor: cluster; between-subject factor: OSA group) and post hoc tests. To examine associations with OSA severity, individual SWA decay was again regressed against apnea–hypopnea index (AHI) within each severity group, reporting decay slope estimates, R^2^, and *p*-values. Differences in the decay between OSA severity groups were compared using *F*-test or analysis of covariance (ANCOVA). Slopes of slow waves in the first and last hour of NREM sleep in the four groups was assessed with two-way ANOVA with appropriate post hoc tests for multiple comparisons.

The association between WASO and decay of SWA was examined using linear regression across all participants (Panel A). The regression model estimated the slope (β), coefficient of determination (R^2^), and significance using an *F*-test. To assess whether this relationship differed by OSA severity, an ANCOVA was performed with WASO as a covariate and OSA group (no OSA, mild, moderate, severe) as a factor, including the group × WASO interaction. Post hoc pairwise comparisons of decay slopes were conducted using linear contrasts when the interaction was significant.

Finally, associations of decay of SWA and slope of slow waves with clinical and PSG variables of interest were assessed using univariable and multivariable linear regression models. Age, sex, and body mass index (BMI) were the covariates used in the multivariable model fit. Separate models were fit for two dependent variables: decay of SWA and the mean slope of slow waves across NREM sleep. All analyses were conducted in GraphPad Prism (version 10.5), MATLAB, and R (version 4.2) for statistical computing at 5% significance level.

## Results

Participants’ apnea severity was categorized based on American Academy of Sleep Medicine scoring criteria (3% desaturation): no OSA-AHI < 5 (*n* = 430), mild OSA-AHI 5–15 (*n* = 267), moderate OSA-AHI 15–30 (*n* = 120), and severe OSA-AHI > 30 (*n* = 128). Differences in demographic, clinical conditions, medication/caffeine/alcohol use, and sleep characteristics across the four groups are shown in Table 1. The proportion of males was higher in all OSA groups. BMI was higher with increasing apnea severity (*p* < 0.001). Cardiometabolic comorbidities, including hypertension, diabetes, and use of antihypertensive, cholesterol-lowering, and diabetes medications, also were progressively greater with increasing OSA severity (all *p* < 0.01). Sleep architecture showed worse fragmentation with increasing OSA severity: total sleep time and sleep efficiency decreased (*p* < 0.001), WASO increased (*p* < 0.001), and N1 percentage increased sharply across groups (*p* < 0.001), while N3 (*p* = 0.026) and REM sleep percentages decreased (*p* < 0.001). As expected, markers of respiratory disturbance (AHI) and ODI increased with increasing apnea severity (*p* < 0.0001 for both). Mean normalized SWA was not different between the groups (*p* = 0.999). The mean decay slope of SWA was progressively less steep or flatter with increasing OSA severity (*p* < 0.001) (Table 1).

**Table 1:** Participant Characteristics by OSA severity (*N* = 945). Data reported as mean (SD), median (IQR), or N (%). *P*-values from ANOVA, Kruskal–Wallis, chi-square, or Fisher’s exact tests. Holm *P*-values are adjusted for multiple comparisons using Holm’s method

Variable	Total *N* = 945	No OSA (*n* = 430)	Mild OSA (*n* = 267)	Moderate OSA (*n* = 120)	Severe OSA (*n* = 128)	*P*-value	Adj P
Age—yr	55.6 (7.8)	54.1 (7.6)	56.3 (7.4)	57.5 (7.5)	57.8 (8.2)	< 0.001	< 0.001
Sex—male	496 (52.5%)	184 (42.8%)	155 (58.1%)	74 (61.7%)	83 (64.8%)	< 0.001	< 0.001
BMI	31.4 (6.7)	28.5 (5.2)	32.4 (6.5)	33.8 (6.3)	36.4 (7.7)	< 0.001	< 0.001
Hypertension—yes	288 (30.5%)	98 (22.8%)	81 (30.3%)	43 (35.8%)	66 (51.6%)	< 0.001	< 0.001
Stroke—yes	12 (1.3%)	6 (1.4%)	1 (0.4%)	0 (0%)	5 (3.9%)	0.027	0.404
Diabetes—yes	83 (8.8%)	21 (4.9%)	21 (7.9%)	14 (11.7%)	27 (21.1%)	< 0.001	< 0.001
Emphysema—yes	12 (1.3%)	4 (0.9%)	3 (1.1%)	3 (2.5%)	2 (1.6%)	0.452	1
Drug: antihistamine—yes	68 (7.2%)	31 (7.2%)	22 (8.2%)	9 (7.5%)	6 (4.7%)	0.646	1
Drug: decongestant—yes	45 (4.8%)	18 (4.2%)	13 (4.9%)	9 (7.5%)	5 (3.9%)	0.473	1
Drug: anti-anxiety—yes	71 (7.5%)	35 (8.1%)	16 (6.0%)	10 (8.3%)	10 (7.8%)	0.738	1
Drug: antidepressant—yes	187 (19.8%)	88 (20.5%)	43 (16.1%)	26 (21.7%)	30 (23.4%)	0.29	1
Drug: asthma treatment—yes	46 (4.9%)	17 (4.0%)	16 (6.0%)	5 (4.2%)	8 (6.2%)	0.54	1
Drug: cholesterol lowering—yes	220 (23.3%)	66 (15.3%)	68 (25.5%)	36 (30.0%)	50 (39.1%)	< 0.001	< 0.001
Drug: diabetes treatment—yes	63 (6.7%)	16 (3.7%)	17 (6.4%)	10 (8.3%)	20 (15.6%)	< 0.001	0.001
Drug: antihypertensive—yes	325 (34.4%)	111 (25.8%)	94 (35.2%)	50 (41.7%)	70 (54.7%)	< 0.001	< 0.001
Drug: thyroid treatment—yes	76 (8.0%)	30 (7.0%)	27 (10.1%)	7 (5.8%)	12 (9.4%)	0.347	1
Narcotics—yes	19 (2.0%)	8 (1.9%)	4 (1.5%)	4 (3.3%)	3 (2.3%)	0.609	1
Sedatives—yes	67 (7.1%)	37 (8.6%)	13 (4.9%)	9 (7.5%)	8 (6.2%)	0.3	1
Stimulants—yes	14 (1.5%)	8 (1.9%)	1 (0.4%)	3 (2.5%)	2 (1.6%)	0.225	1
Alcohol consumption						0.865	1
No Alcohol	323 (34.2%)	149 (34.7%)	87 (32.6%)	43 (35.8%)	44 (34.4%)		
Moderate	551 (58.3%)	248 (57.7%)	164 (61.4%)	66 (55.0%)	73 (57.0%)		
Heavy	71 (7.5%)	33 (7.7%)	16 (6.0%)	11 (9.2%)	11 (8.6%)		
Daily caffeine						0.294	1
No Caffeine	159 (16.8%)	74 (17.2%)	46 (17.2%)	18 (15.0%)	21 (16.4%)		
1–4 beverages	638 (67.5%)	302 (70.2%)	177 (66.3%)	77 (64.2%)	82 (64.1%)		
> 4 beverages	148 (15.7%)	54 (12.6%)	44 (16.5%)	25 (20.8%)	25 (19.5%)		
Total sleep—minutes	368.2 (58.4)	376.2 (57.3)	367.6 (56.4)	361.6 (60.8)	348.4 (58.9)	< 0.001	< 0.001
N1 percent	8.4 (5.9–12.4)	7.8 (5.8–11.1)	8.1 (5.5–12.2)	9.1 (6.3–13.8)	11.5 (7.7–18.2)	< 0.001	< 0.001
N2 percent	69.3 (8.2)	68.5 (8.3)	69.6 (7.5)	70.0 (7.5)	70.8 (9.6)	0.019	0.304
N3 percent	1.7 (0.2–5.8)	2.3 (0.3–6.5)	1.7 (0.1–6.0)	1.1 (0.1–4.5)	1.0 (0.0–4.4)	0.002	0.026
REM percent	16.7 (6.0)	17.9 (5.9)	17.1 (5.2)	16.4 (6.4)	12.4 (5.7)	< 0.001	< 0.001
Sleep Efficiency	80.7 (10.0)	82.3 (9.8)	80.5 (10.0)	79.8 (9.1)	76.5 (9.7)	< 0.001	< 0.001
WASO—minutes	43.3 (24.8–68.6)	36.9 (21.9–58.8)	44.4 (25.5–69.8)	50.5 (30.4–71.7)	56.8 (36.3–78.6)	< 0.001	< 0.001
ODI per hour	1.2 (0.4–3.2)	0.5 (0.2–0.9)	1.8 (0.8–2.9)	3.5 (1.9–5.4)	8.0 (5.0–14.9)	< 0.001	< 0.001
Mean SWA	5.3 (2.0)	5.4 (2.0)	5.3 (1.9)	5.0 (1.9)	5.4 (2.1)	0.352	1
Decay of SWA	− 0.4 (0.4)	− 0.5 (0.4)	− 0.4 (0.4)	− 0.3 (0.4)	− 0.2 (0.5)	< 0.001	< 0.001
Mean slope of slow waves	216.7 (25.7)	219.3 (26.5)	215.0 (25.0)	214.3 (24.2)	214.2 (25.4)	0.048	0.67

Values reflect available data. *OSA* obstructive sleep apnea, *ODI* oxygen desaturation index, *SWA* slow wave activity, *REM* rapid eye movement, *WASO* wake after sleep onset. *Adj P* Bonferroni-Holm adjusted *p*-values

### Sleep Homeostasis measures

#### A: Decay of SWA

**Fig. 2:**
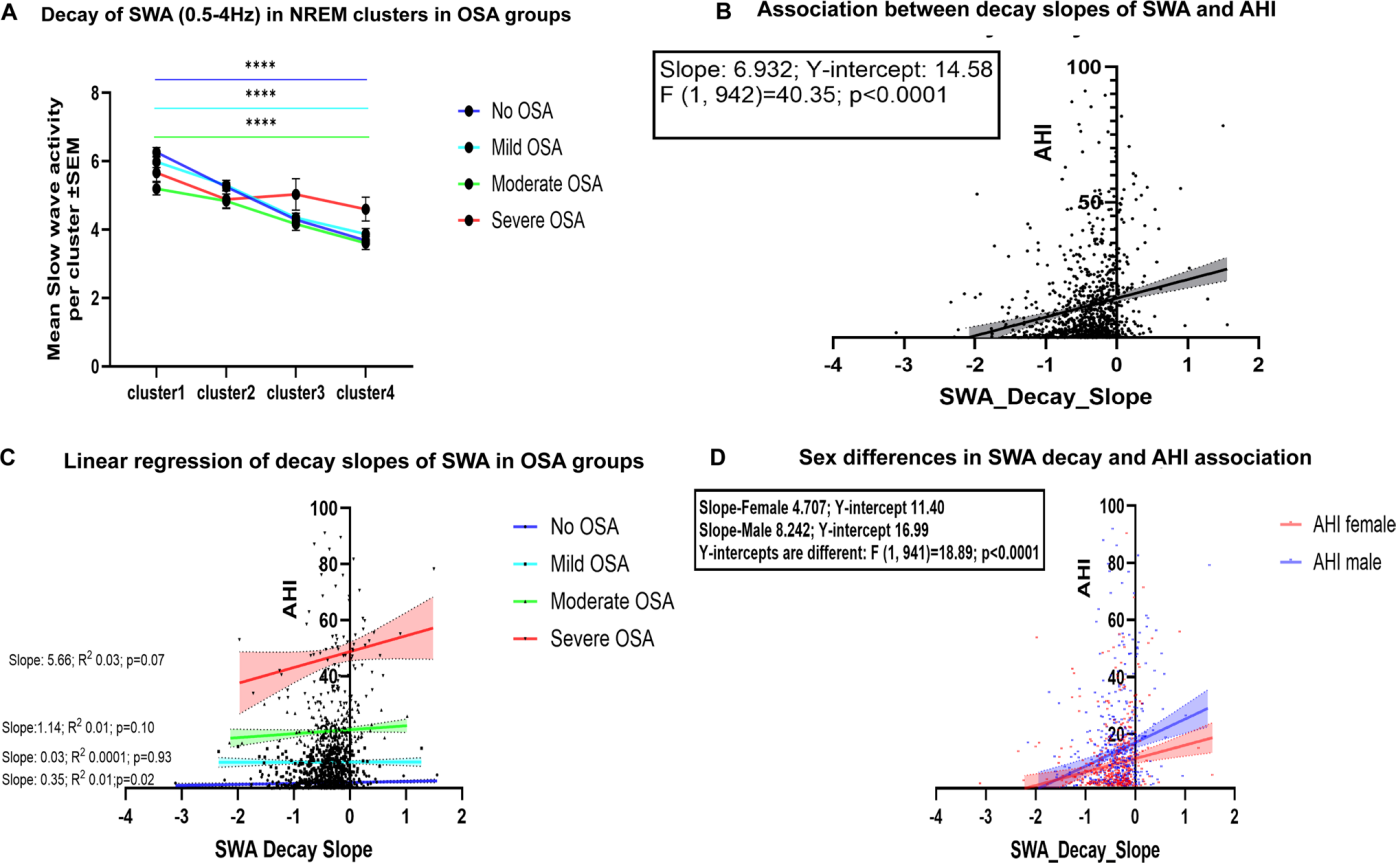
**A** Mean normalized SWA in each of the 4 NREM clusters in no OSA (blue), mild OSA (cyan), moderate OSA (green), and severe OSA (red) with error bars representing SEM. Significant group differences indicated with horizontal bars (**** *p* < 0.0001). **B** Association between SWA decay and AHI across all subjects. Solid line is the linear regression fit and the shaded area represents 95% confidence interval. **C** Scatter plots with fitted regression lines and 95% confidence bands show the association between AHI and SWA decay for four groups: no OSA, mild OSA (cyan), moderate OSA, and severe OSA. Scatter plots show individual data points for SWA decay (*x*-axis) versus AHI (*y*-axis). Colored regression lines represent linear fits for each group: no OSA-blue; mild OSA-cyan; moderate OSA-green; severe OSA-red. Shaded areas represent 95% confidence intervals. **D** Sex differences in association of SWA decay and AHI. Regression lines for females (red) and males (blue) with corresponding 95% confidence intervals are shown

We examined how mean SWA decayed across the night in successive NREM clusters. Two-way ANOVA showed an interaction of group × time (F (8.771, 22,163) = 5.51; *p* < 0.0001) as well as a main effect of group (F (3, 938) = 2.772; *p* < 0.04) and time (F (2.92, 2163) = 105.9; *p* < 0.0001). Post hoc analyses showed that SWA was significantly lower in fourth compared to first NREM cluster in no OSA, mild and moderate OSA groups (p < 0.0001 for each) but not in the severe OSA group (*p* = 0.051). Comparing the mean SWA between the OSA groups, only differences seen were in no OSA vs moderate OSA (6.26 ± 0.14 vs 5.20 ± 0.18; p < 0.0001) and between mild OSA and moderate OSA (5.98 ± 0.16 VS 5.20 ± 0.18; *p *= 0.008) in cluster 1 (Fig. 2A) with no other group differences seen across remaining NREM clusters. These data suggest that SH is impaired in the severe OSA group.

Linear regression showed that the decay of SWA had a positive association with AHI (*β* = 6.93, Y-intercept 14.58; *R*^*2*^ = 0.04; [F(1,942) = 37.85; *p* < 0.0001]) suggesting that with increasing AHI, there was reduced SWA decay (Fig. 2B). There were significant group differences [F(3,936) = 6.45; *p* = 0.0003] with severe OSA group showing the steepest relationship with a trend towards significance, and no OSA group shows a significant but weak association and mild/moderate groups showing no association (Fig. 2C). There were sex differences with males showing significantly steeper slope of SWA compared to females in addition to significant differences in the intercepts (Fig. 2D). These findings suggest that, in severe OSA, overnight decay of SWA is worse with increasing AHI and that impairments are likely greater in males.

#### B: Relationship between WASO and SWA

**Fig. 3:**
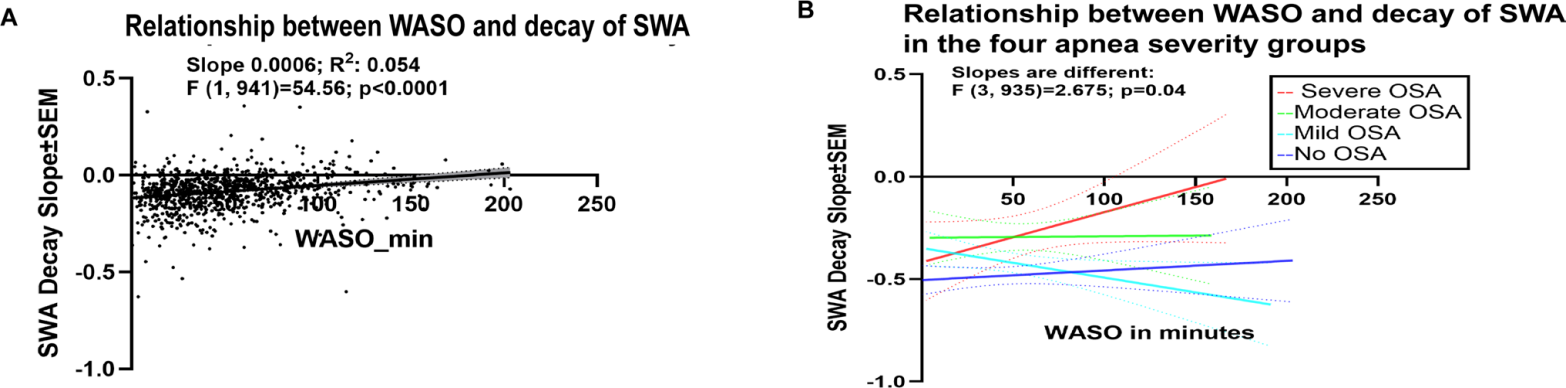
**A** Scatter plot showing the association between wake after sleep onset (WASO, minutes) and SWA decay (mean ± SEM) across all participants along with the regression line and 95% confidence intervals. **B** Linear regression of SWA decay versus WASO stratified by OSA severity groups: no OSA (blue), mild OSA (cyan), moderate OSA (green), and severe OSA (red). Shaded areas represent 95% confidence intervals

Examining relationship between total WASO during the night and SWA decay, higher WASO was associated with slower or flatter decay of SWA. The fitted regression line indicates a small positive relationship (*β* = 0.0006, *R*^*2*^ = 0.054; F(1,941) = 54.56; *p* < 0.0001) (Fig. 3A). We then tested the effect of WASO on SWA decay in each OSA group. ANCOVA revealed that slopes of decay differ significantly among groups (F(3,935) = 2.675; *p* = 0.04). Pairwise contrasts of slopes indicate that severe OSA differs significantly from no OSA (*p* = 0.02) and mild OSA (*p* = 0.04), while differences between mild and moderate OSA are not significant (*p* = 0.10) (Fig. 3B).

#### C: Decay of slope of slow wave

**Fig. 4:**
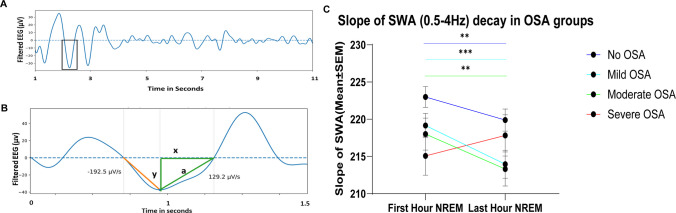
**A** Example of a negative slow wave captured from EEG. **B** Closer look at the negative slow wave and demonstrate how the slow wave slope is calculated (slope (a) = abs(y/x)). **C** Change in slopes of slow waves (0.5–4 Hz) from the first and last hour of NREM sleep in (OSA) severity groups. Mean ± SEM are shown for participants classified as no OSA (blue), mild OSA (cyan), moderate OSA (green), and severe OSA (red)

A final measure of SWA homeostasis was the change in slope of slow wave (Fig. 4A and B) from the first to the last hour of NREM sleep. Two-way ANOVA revealed a significant interaction between apnea severity and time (F(3,940) = 3.52; *p* = 0.01), as well as main effects of time (F(1,940) = 9.91; *p* = 0.001) and apnea group (F(3,942) = 2.92; *p* = 0.03). Post hoc Tukey tests showed no between-group differences in slopes of slow waves at either time point (*p* > 0.05). However, slopes decreased significantly from the first to last hour in no OSA (*p* = 0.0002), mild OSA (*p* = 0.0001), and moderate OSA (*p* = 0.009), but not in severe OSA (*p* = 0.20) (Fig. 4C). These findings indicate that severe OSA exhibited the greatest disruption of this homeostatic measure, with no significant change in slopes of slow waves across the night.

### Clinical predictors of the SH measures

In univariable analyses, moderate and severe OSA had a larger SWA decay on average compared to no OSA participants; this trend was slightly muted but still statistically significant in the multivariable model. Other factors that were significantly associated with SWA decay after accounting for age, sex, and BMI included antidepressant medication use (β [95% CI] = − 0.079 [− 0.149, − 0.008]; *p* = 0.029), sedative medication use (0.109 [0.002, 0.215]; *p* = 0.045), daily caffeine intake > 4 beverages (0.118 [0.022, 0.215]; *p* < 0.001) compared to no caffeine intake, total sleep time (− 0.056 [− 0.085, − 0.026] per additional hour of sleep; *p* < 0.001), N3 sleep categories compared to 0–2% (all *p* < 0.01), log transformed WASO (*p* < 0.001), and ODI per hour ≥ 15 (0.175 [0.020, 0.330]; *p* = 0.027) compared to < 5 ODI per hour. Hypertension, diabetes, antihistamine use, anti-anxiety medication use, cholesterol lowering agent use, antihypertensive medication use, thyroid treatment medication use, and alcohol use were not significantly associated with SWA decay (Table 2).

**Table 2:** Results of univariable and multivariable regression analysis of possible associated variables with SWA Decay Slope. *WASO* wake after sleep onset, *ODI* oxygen desaturation index)

Variable	Univariable Coef (95% CI)	*P*-value	Multivariable Coef (95% CI)	*P*-value
OSA severity				
No OSA	ref	–-	ref	–-
Mild OSA	0.061 (− 0.005–0.127)	0.068	0.017 (− 0.052–0.086)	0.632
Moderate OSA	0.168 (0.081–0.254)	< 0.001	0.106 (0.014–0.198)	0.024
Severe OSA	0.234 (0.149–0.318)	< 0.001	0.157 (0.062–0.252)	0.001
Hypertension				
No	ref	–-	ref	–-
Yes	0.076 (0.015–0.136)	0.014	0.023 (− 0.040–0.086)	0.471
Diabetes				
No	ref	–-	ref	–-
Yes	0.106 (0.008–0.204)	0.035	0.043 (− 0.056–0.142)	0.393
Antihistamine medication use				
No	ref	–-	ref	–-
Yes	0.008 (− 0.100–0.116)	0.89	0.033 (− 0.073–0.139)	0.544
Anti-anxiety medication use				
No	ref	–-	ref	–-
Yes	− 0.037 (− 0.143–0.068)	0.487	− 0.002 (− 0.106–0.102)	0.965
Antidepressant medication use				
No	ref	–-	ref	–-
Yes	− 0.112 (− 0.182– − 0.043)	0.002	− 0.079 (− 0.149– − 0.008)	0.029
Cholesterol lowering medication use				
No	ref	–-	ref	–-
Yes	0.003 (− 0.063–0.069)	0.941	− 0.066 (− 0.133–0.001)	0.055
Antihypertensive medication use				
No	ref	–-	ref	–-
Yes	0.089 (0.031–0.148)	0.003	0.029 (− 0.033–0.091)	0.360
Hypothyroidism treatment				
No	ref	–-	ref	–-
Yes	− 0.051 (− 0.153–0.052)	0.33	− 0.049 (− 0.151–0.053)	0.342
Sedative medication use				
No	ref	–-	ref	–-
Yes	0.079 (− 0.030–0.188)	0.154	0.109 (0.002–0.215)	0.045
Alcohol consumption				
No alcohol	ref	–-	ref	–-
Moderate	− 0.005 (− 0.065–0.055)	0.879	− 0.006 (− 0.065–0.054)	0.852
Heavy	0.042 (− 0.071–0.154)	0.468	0.049 (− 0.062–0.159)	0.388
Daily caffeine use				
No caffeine	ref	–-	ref	–-
1–4 beverages	0.020 (− 0.056–0.095)	0.609	0.015 (− 0.059–0.089)	0.686
> 4 beverages	0.140 (0.042–0.237)	0.005	0.118 (0.022–0.215)	0.016
Total sleep time	− 0.080 (− 0.108– − 0.052)	< 0.001	− 0.056 (− 0.085– − 0.026)	< 0.001
N3 sleep category				
0–2%	ref	–-	ref	–-
2–7%	− 0.114 (− 0.178– − 0.050)	< 0.001	− 0.098 (− 0.162– − 0.034)	0.003
7–12%	− 0.241 (− 0.325– − 0.156)	< 0.001	− 0.211 (− 0.296– − 0.126)	< 0.001
> = 12%	− 0.350 (− 0.450– − 0.250)	< 0.001	− 0.319 (− 0.420– − 0.218)	< 0.001
WASO	0.137 (0.103–0.170)	< 0.001	0.111 (0.076–0.147)	< 0.001
ODI per hour				
< 5	ref	–-	ref	–-
5–15	0.100 (0.017–0.184)	0.019	0.060 (− 0.026–0.145)	0.170
> = 15	0.271 (0.118–0.425)	0.001	0.175 (0.020–0.330)	0.027

^*^Total Sleep Time estimates per 1 h change

^**^WASO log transformed

^Multivariable adjusted for Age, Sex, BMI

To further characterize the relationship between antidepressant and sedative use and SWA decay, we examined each class of drugs used as monotherapy (as reported by the participants) using univariable and multivariable models adjusting for age, sex, and BMI. In univariable analyses, SNRIs (β [95% CI] = − 0.393 (− 0.651– − 0.135) *p* = 0.003) and TCAs (− 0.289 [− 0.492, − 0.087); *p* = 0.005) were associated with flatter SWA decay or impaired SH, compared with individuals not taking antidepressants. In univariate analysis only other class that had a significant association with decay of SWA was SSRIs (β [95%CI] = − 0.104 (− 0.203– − 0.004); *p* = 0.04). In multivariable models, the association persisted for TCAs (− 0.268 [− 0.466, − 0.069]; *p* = 0.008) and SNRIs (− 0.381 (− 0.634– − 0.129); *p* = 0.003). The effects of SSRIs (*β* = − 0.080; *p* = 0.114), bupropion (*β* = − 0.026; *p* = 0.78), SARIs (*β* = 0.24; *p* = 0.16), and benzodiazepines (*β* = 0.224; *p* = 0.08) were not statistically significant in multivariable analysis (Table 3). These findings suggest that TCAs and SNRIs exert the strongest independent effect in impairing SH, whereas other antidepressant or antianxiety classes show no significant associations after controlling for covariates.

**Table 3:** Results of univariable and multivariable ANOVA of possible associated antidepressant and antianxiety medication variables with decay of SWA along with sample sizes (*SSRI* selective serotonin receptor inhibitor, *SNRI* serotonin and norepinephrine reuptake inhibitor, *SARI* serotonin antagonist and reuptake inhibitor, *TCA* tricyclic antidepressant, *BZD* benzodiazepine)

	Univariate analysis		Multivariable analysis^	
Variable	Coef (95% CI)	*P* value	Coef (95% CI)	*P* value
Drug/mechanism				
None (742)	ref	–-	ref	ref
SSRI *n* = 81)	− 0.104 (− 0.203– − 0.004)	0.041	− 0.080 (− 0.178–0.019)	0.114
Wellbutrin (*n* = 20)	− 0.092 (− 0.285–0.100)	0.348	− 0.026 (− 0.215–0.163)	0.789
TCA (*n* = 18)	− 0.289 (− 0.492– − 0.087)	0.005	− 0.268 (− 0.466– − 0.069)	0.008
SNRI (*n* = 11)	− 0.393 (− 0.651– − 0.135)	0.003	− 0.381 (− 0.634– − 0.129)	0.003
BZD (*n* = 11)	0.234 (− 0.024–0.492)	0.075	0.224 (− 0.029–0.476)	0.083
SARI (*n* = 6)	0.210 (− 0.138–0.558)	0.237	0.241 (− 0.100–0.583)	0.166

^Multivariable analysis controls for age, sex, and BMI as covariates

3 patients removed due to sample sizes of *N* = 1 for MAO1, barbiturates, and non-BZD hypnotic individually

Finally, we examined predictors of the mean slope of slow wave across all NREM sleep. Univariable analyses showed that multiple clinical factors were associated with lower slope of slow wave. These included mild and severe OSA, hypertension, diabetes, use of anti-anxiety medications, antidepressants, cholesterol-lowering agents, antihypertensives, and both moderate and high caffeine intake. After adjustment for age, sex, and BMI, OSA severity was no longer associated with slope of slow wave, whereas diabetes (β [95% CI] = − 9.1 [− 14.9, − 3.4]; *p* = 0.002), anti-anxiety medications (− 11.7 [− 17.8, − 5.7]; *p* < 0.001), antidepressants (− 10.3 [− 14.4, − 6.2]; *p* < 0.001), antihypertensive medications (− 4.2 [− 7.8, − 0.6]; *p* = 0.021), sedatives (− 7.1 [− 13.4, − 0.9]; *p* = 0.024), and high caffeine intake (> 4 beverages/day; − 10.9 [− 16.5, − 5.2]; *p* < 0.001) compared to no caffeine intake remained significant predictors of a lower or flatter slope of slow wave. In contrast, the proportion of N3 sleep demonstrated a robust graded association with slope of slow wave, with N3 levels of 2–7%, 7–12%, and ≥ 12% corresponding to progressively higher slopes of slow waves (*β* = + 8.1, + 13.1, and + 28.2, respectively; all *p* < 0.001). Total sleep time, WASO, and ODI per hour classification were not significantly associated with slope of slow wave after adjustment (Table S1).

## Discussion

In this large, population-based cohort, we found that multiple components of SH were disrupted with increasing OSA severity. Although average SWA did not differ between groups, individuals with more severe OSA demonstrated markedly impaired overnight SWA dynamics, reflected by flatter SWA decay across NREM sleep, and diminished decline in slow wave slopes across the night. Moreover, the severe OSA group, which showed little to no reduction in SWA or slow-wave slope from the first to the last NREM episode, indicated blunted dissipation of sleep pressure. Beyond respiratory event severity, several clinical factors—including diabetes, antihypertensive, antidepressant, anti-anxiety and sedative medication use, and high caffeine intake—were independently associated with impaired SWA decay, highlighting that sleep homeostasis disruption in OSA is influenced by a broader constellation of metabolic and pharmacologic factors. In contrast, greater N3 sleep percentage showed a strong, graded association with steeper SWA decay and steeper slow-wave slopes, supporting the protective role of deep sleep in maintaining homeostatic regulation. Together, these findings suggest that OSA is associated with multidimensional impairments in sleep homeostasis, with the greatest disruptions occurring in severe disease and in individuals with additional metabolic or medication-related risk factors.

Our data are consistent with a previous study that reported reduction in overnight dissipation of SWA in patients with OSA compared with those without [24]. SWA dissipates more slowly across the night in OSA, suggesting impaired SH, thought to be due to sleep fragmentation [26–28]. High-density EEG studies have demonstrated that SWA exhibits regional variability in OSA, with frontal dominance and altered topographical distribution compared to controls [29]. We observed that severe OSA markedly blunted overnight SWA decay. There were no sex differences in decay of SWA reported previously, but our data shows that males exhibited higher AHI values for the same SWA decay level, suggesting a sex-related vulnerability. Furthermore, whereas prior short-term work has emphasized the relative stability of quantitative SWA measures across nights [30], our one-night analyses indicate that severe OSA is associated with attenuated overnight SWA decay and a reduced decline in slow-wave slope from the first to the last hour of NREM sleep.

Our univariate and multivariate models suggested some polysomnography variables and some medical conditions impacted SWA decay and slopes of slow waves. Not all studies have reported changes in the dynamics of SWA in OSA. One study observed higher absolute delta power across the night in OSA patients compared with controls [31], and it was also noted that post-apneic arousals can contribute to higher SWA by another [27] suggesting that the alterations in SH in OSA may vary by methodology or patient characteristics. These data underscore the complexity of SWA regulation in OSA and highlight the need for standardized spectral analysis and consideration of disease severity, sex, and comorbidities in future research. Most of the prior studies examined dynamics of absolute delta power and did not use any other measures of SH [26–30]. Thus, prior studies used SWA as the main electrophysiologic marker of SH, but SH represents a dynamic regulatory process, characterized not just by absolute SWA levels, but by how sleep pressure is accumulated, dissipated, downscaled, and re-expressed in response to perturbations across the night. Our data more thoroughly show how each of these dynamic processes of SH are impacted by severity of OSA.

Impaired SH in OSA may have profound impact on clinically relevant outcomes. SH is critical for synaptic downscaling, memory consolidation, and neuroplasticity [14]. Disruption of SH reflected in SWA dynamics has been linked to deficits in attention, executive function, and working memory in OSA patients [32, 33]. Furthermore, reduced SWA and fragmented NREM sleep are associated with increased risk of Alzheimer’s disease and other neurodegenerative disorders, likely through impaired glymphatic clearance and accumulation of β-amyloid [34, 35]. Levels of Aβ decreased and absolute SWA increased after treatment with CPAP in one study. Beyond cognition, OSA-related alterations in sleep architecture predict higher all-cause and cardiovascular mortality, independent of traditional risk factors [3, 36]. Disruption in overnight dissipation of SWA also has implications for all-cause mortality as shown in a recent longitudinal study [6]. Similarly, another recent study showed that impaired SH characterized by loss of overnight decay of SWA in those that succumbed to sudden unexpected death in epilepsy (SUDEP) [37]. Our findings of attenuated overnight SWA dissipation and changes in slopes of slow waves in severe OSA suggest a mechanistic pathway linking sleep fragmentation to neurocognitive decline and increased mortality risk. These observations underscore the importance of targeting both respiratory disturbances and SH in therapeutic strategies for OSA.

Our data further suggest that comorbid conditions and medication exposures influence SH beyond the effects attributable to OSA severity alone. Specifically, percent N3 sleep and sedative medication use were associated with relatively preserved SH, characterized by a steeper overnight decay of SWA. In contrast, antidepressant use as a composite group and time in WASO were associated with impaired SH, reflected by a flatter SWA decay. Prior studies examining the effects of antidepressants on SWA and delta power have documented class-specific modulation of slow wave expression and homeostatic dynamics. For example, tricyclic antidepressants such as amitriptyline, trimipramine, and clomipramine have been shown to enhance increase slow wave sleep and increases in SWA [38–40]. Serotonergic agents including fluoxetine and paroxetine tend to reduce SWA and blunt homeostatic SWA responses [41, 42]. Data with other antidepressants show either increased, reduced SWA, or no change [41, 43, 44]. In this cohort, antidepressant use was consistently associated with alterations in SWA dynamics, reflected by both a flatter overnight decline in SWA and reduced mean slow-wave slopes, findings indicative of impaired SH. Notably, among the different antidepressant classes, TCAs and SNRIs showed the strongest and most robust association with disrupted SWA decay even after adjusting for covariates, suggesting a class-specific influence on homeostatic sleep regulation. Interestingly, sleep in depression is shown to be characterized by impaired slow wave homeostasis [45]. These results underscore the need for future studies to incorporate measures of antidepressant exposure and depression severity when measuring SH. Moreover, targeted evaluation of whether concurrent use of multiple antidepressant classes or higher cumulative medication burden affects additional sleep physiology markers (e.g., intra-night SWA dynamics, spectral power profiles, or slow-wave morphology) will be essential in larger, purpose-designed samples to parse medication effects from disease-related alterations in sleep homeostasis.

This study has several limitations. First, the analysis was based on observational data from a single cohort, which limits causal inference and may introduce confounders despite multivariate adjustments. Second, SWA was derived from standard EEG leads rather than high-density EEG, precluding detailed assessment of regional SWA changes that may provide additional mechanistic insights. Medication use and comorbidities were self-reported and may be subject to misclassification which is a potential limitation. We had a minority of subjects with ODI > 30 and hence we limited the assessment of impact of ODI on decay of SWA to subjects whose ODI was > 15 and this is another limitation. We did not compare SH measures before and after CPAP initiation, which limits understanding of reversibility and treatment effects. The study cohort was predominantly white, with limited representation of other racial and ethnic groups, which may restrict the generalizability of these findings to more diverse populations. Finally, the cohort predominantly included middle-aged adults, limiting generalizability to younger or older populations. Future studies using longitudinal designs, high-density EEG, and mechanistic biomarkers are needed to confirm these findings and clarify their clinical implications.

In summary, this study systematically demonstrates that increasing OSA is associated with impairment of SH in a large community. Severe OSA showed a markedly blunted overnight decay of SWA and a reduced decline in slow-wave slope from the beginning to the end of the night, suggesting disruption of fundamental sleep restorative processes. The impaired SH measures reflect incomplete dissipation of accumulated sleep pressure that could manifest as nonrestorative sleep. The intranight sleep fragmentation from OSA likely undermines the compensatory homeostatic processes. Future studies should take disease duration into account and determine whether the impairments in SH are progressively worsen over time with chronicity of OSA. Impaired SH may compromise synaptic renormalization and brain energy balance by preventing adequate downscaling of wake-potentiated synapses, thereby sustaining excessive synaptic load, metabolic demand, and reduced capacity for learning-related plasticity. These findings provide mechanistic insight into how OSA may contribute to neurocognitive decline, metabolic dysregulation, and increased mortality risk. Future studies should address whether CPAP therapy completely reverses impairments in SH caused by OSA. Finally, studies in diverse populations and across different age groups are essential to improve generalizability and inform personalized treatment strategies.

## Supplementary Information

Below is the link to the electronic supplementary material.

ESM 1 (PDF 160 KB)

## Data Availability

All polysomnography data from Wisconsin Sleep Cohort are available as part of the National Sleep Research Resource and can be directly obtained by communicating (support@sleepdata.org). The Python and MATLAB scripts used for analysis will be made available upon reasonable request to the corresponding author.
