# Supplementary material for: Sleep homeostasis impairment across obstructive sleep apnea severity: findings from a population-based cohort study
Source: J Clin Sleep Med. 2026 Aug 4;22(1):134. doi: 10.1007/s44470-026-00131-6 (PMC13438536; doi:10.1007/s44470-026-00131-6)
Supplement: Supplementary file 1 [file 44470_2026_131_MOESM1_ESM.pdf]

Supplementary Material:

Table of Contents:

1. **Page 1: Table S1**

Table S1: Generalized linear model fixed coefficients of predictors of mean slope of slow waves from all NREM sleep.

Variable	Univariable Coef (95% CI)	P-value	Multivariable Coef (95% CI)	P-value
**OSA Severity:**				
No OSA	ref.	---	ref.	---
Mild OSA	-4.3 (-8.3–-0.4)	0.03	-0.9 (-4.9–3.2)	0.681
Moderate OSA	-5.0 (-10.2–0.2)	0.059	-0.4 (-5.8–5.0)	0.885
Severe OSA	-5.1 (-10.2–-0.1)	**0.047**	0.8 (-4.8–6.4)	0.788
**Hypertension:**				
No	ref.	---	ref.	---
Yes	-4.2 (-7.7–-0.6)	**0.021**	-3.0 (-6.7–0.6)	0.104
**Diabetes:**				
No	ref.	---	ref.	---
Yes	-10.7 (-16.5–-4.9)	**<0.001**	-9.1 (-14.9–-3.4)	**0.002**
**Antihistamine use:**				
No	ref.	---	ref.	---
Yes	4.8 (-1.5–11.2)	0.138	3.3 (-2.9–9.5)	0.29
**Anti-anxiety medication use:**				
No	ref.	---	ref.	---
Yes	-9.1 (-15.3–-2.9)	**0.004**	-11.7 (-17.8–-5.7)	**<0.001**
**Antidepressant medication use:**				
No	ref.	---	ref.	---
Yes	-6.0 (-10.2–-1.9)	**0.004**	-10.3 (-14.4–-6.2)	**<0.001**
**Cholesterol lowering drug use:**				
No	ref.	---	ref.	---
Yes	-5.5 (-9.3–-1.6)	**0.006**	-3.0 (-6.9–0.9)	0.133
**Antihypertensive medication use:**				
No	ref.	---	ref.	---
Yes	-5.7 (-9.1–-2.2)	**0.001**	-4.2 (-7.8–-0.6)	**0.021**
**Hypothyroidism treatment:**				
No	ref.	---	ref.	---
Yes	2.2 (-3.9–8.2)	0.478	-0.5 (-6.5–5.4)	0.864
**Sedative medication use:**				
No	ref.	---	ref.	---
Yes	-4.5 (-10.9–1.8)	0.163	-7.1 (-13.4–-0.9)	**0.024**
**Alcohol Consumption:**				
No Alcohol	ref.	---	ref.	---
Moderate	-2.8 (-6.4–0.7)	0.116	-1.8 (-5.2–1.7)	0.318
Heavy	-3.3 (-9.9–3.4)	0.335	-2.8 (-9.3–3.6)	0.393
**Daily Caffeine use:**				
No Caffeine	ref.	---	ref.	---
1–4 beverages	-4.7 (-9.1–-0.2)	0.039	-4.0 (-8.3–0.3)	0.068
>4 beverages	-13.6 (-19.3–-7.9)	**<0.001**	-10.9 (-16.5–-5.2)	**<0.001**
**Total Sleep Time:**				
TST	2.3 (0.6–4.0)	**0.007**	0.5 (-1.2–2.2)	0.58
N3 sleep category				

0–2%	ref.	---	ref.	---
2–7%	9.9 (6.3–13.5)	<0.001	8.1 (4.5–11.8)	<0.001
7–12%	15.8 (11.0–20.6)	<0.001	13.1 (8.3–17.9)	<0.001
>=12%	31.4 (25.7–37.1)	<0.001	28.2 (22.5–33.9)	<0.001
**WASO**	-0.1 (-2.2–1.9)	0.916	1.9 (-0.2–4.1)	0.072
**ODI per hour**				
<5	ref.	---	ref.	---
5–15	-6.7 (-11.6–-1.7)	**0.008**	-4.3 (-9.2–0.7)	0.095
>=15	-3.0 (-12.1–6.1)	0.515	2.6 (-6.5–11.7)	0.578

* Total Sleep Time estimates per 1 hour change

** WASO log transformed

^ Multivariable adjusted for Age, Sex, BMI
